# Supplementary figures and images for: Cannabinoid receptor 1 positive allosteric modulator ZCZ011 shows differential effects on behavior and the endocannabinoid system in HIV-1 Tat transgenic female and male mice
Source: PLoS One. 2024 Jun 24;19(6):e0305868. doi: 10.1371/journal.pone.0305868 (PMC11195999; doi:10.1371/journal.pone.0305868)

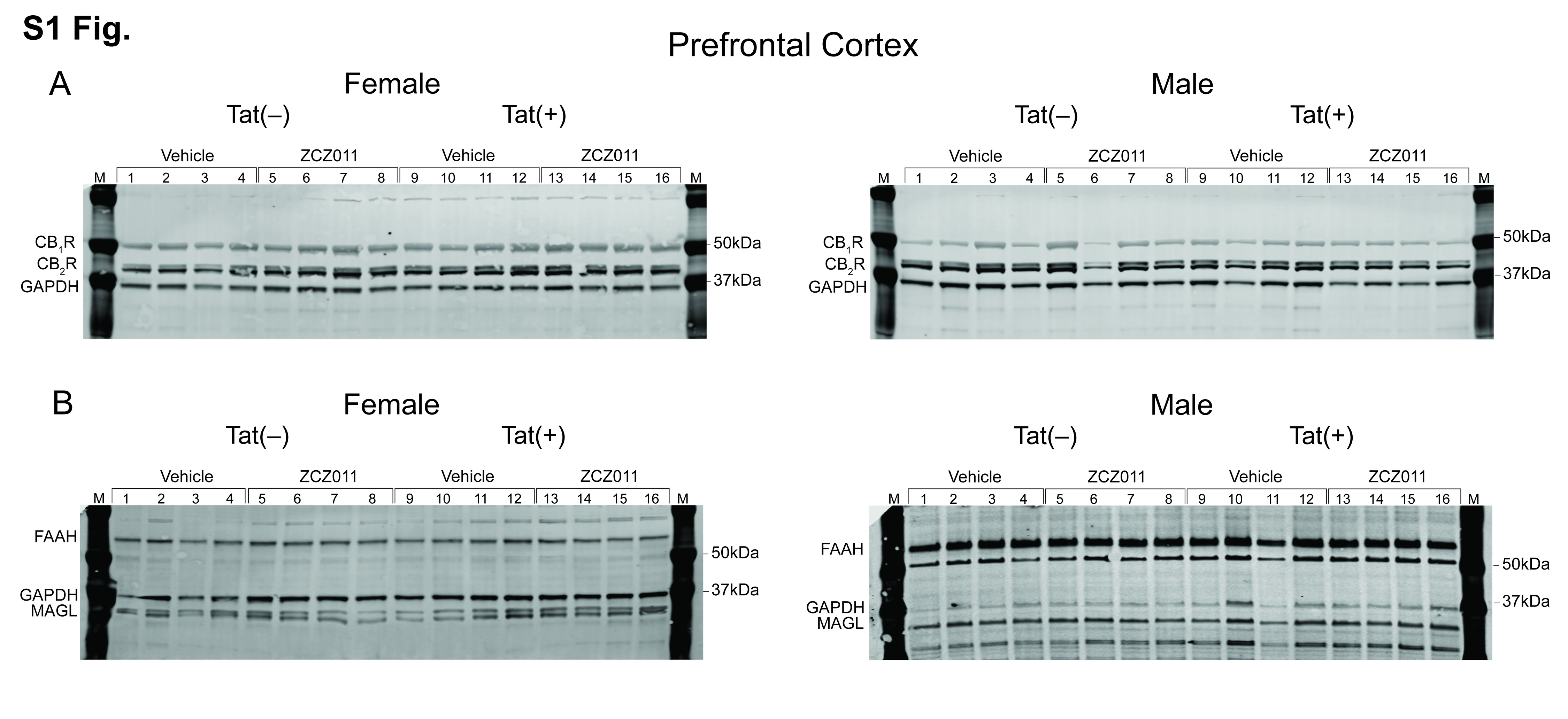

Supplement: S1 Fig — Images show original (A) CB1R, CB2R, and GAPDH and (B) FAAH, MAGL, and GAPDH for females and males. Tat(–) vehicle- and ZCZ011-treated mice are represented by lanes 1–4 and 5–8 respectively. Tat(+) vehicle- and ZCZ011-treated mice are represented by lanes 9–12 and 13–16 respectively. M: molecular weights of marker protein (kDa). (TIF) [file pone.0305868.s001.tif]

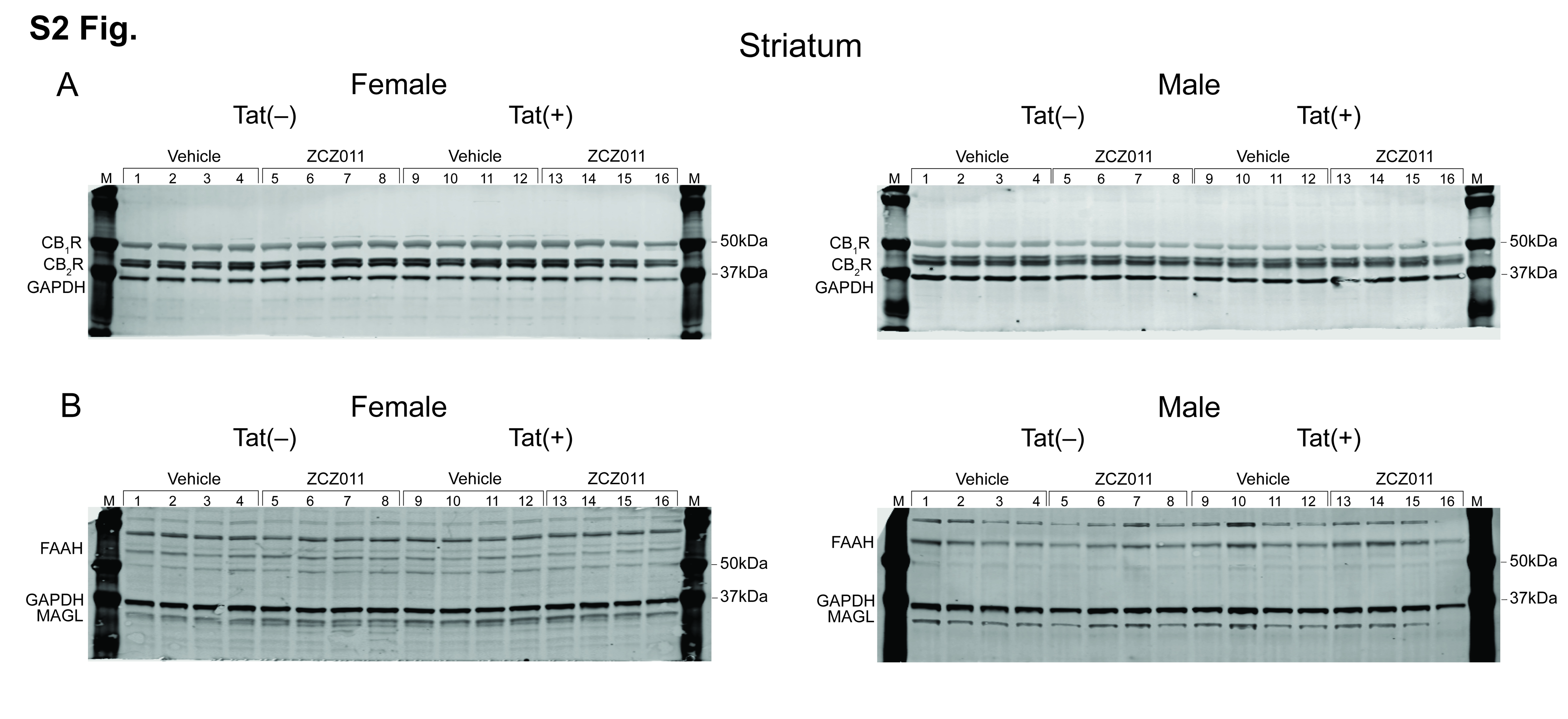

Supplement: S2 Fig — Images show original (A) CB1R, CB2R, and GAPDH and (B) FAAH, MAGL, and GAPDH for females and males. Tat(–) vehicle- and ZCZ011-treated mice are represented by lanes 1–4 and 5–8 respectively. Tat(+) vehicle- and ZCZ011-treated mice are represented by lanes 9–12 and 13–16 respectively. M: molecular weights of marker protein (kDa). (TIF) [file pone.0305868.s002.tif]

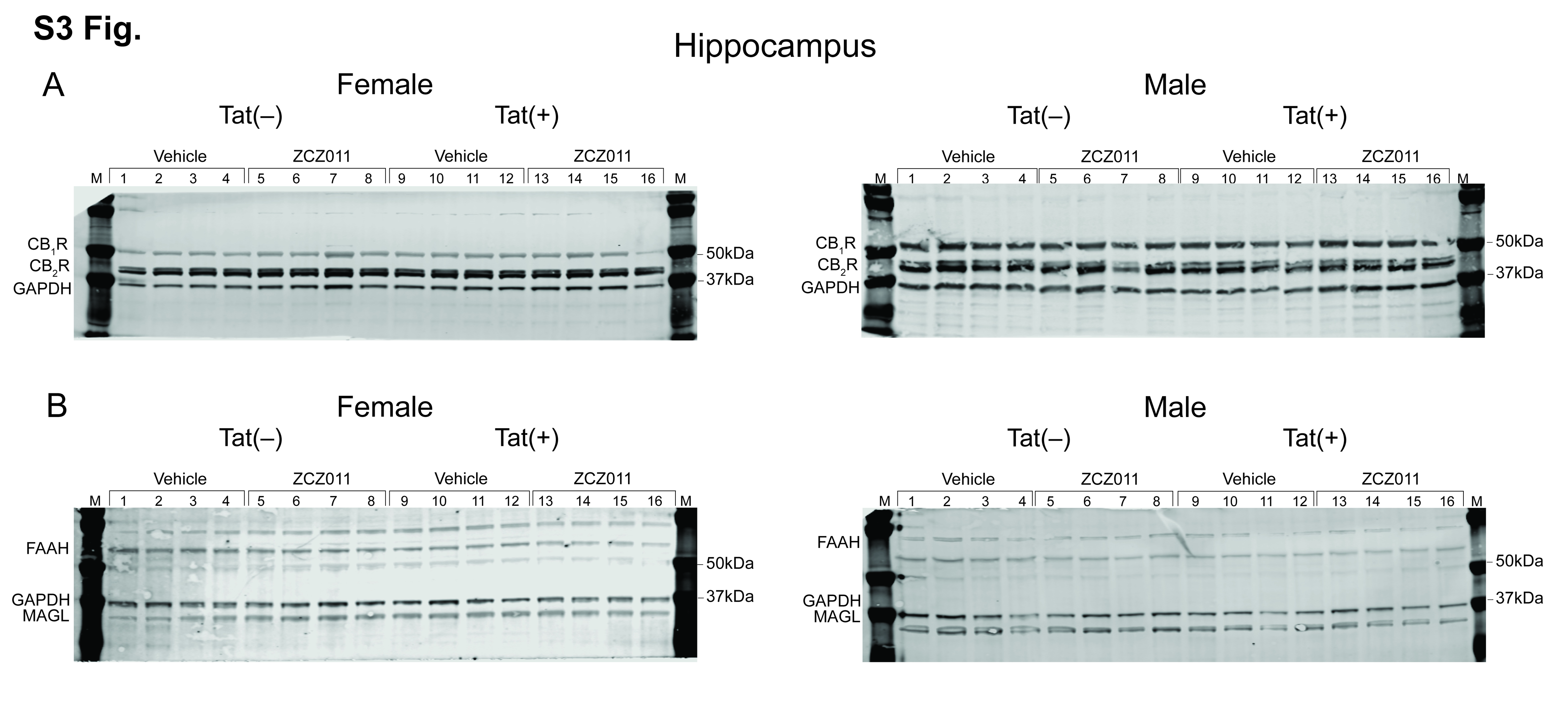

Supplement: S3 Fig — Images show original (A) CB1R, CB2R, and GAPDH and (B) FAAH, MAGL, and GAPDH for females and males. Tat(–) vehicle- and ZCZ011-treated mice are represented by lanes 1–4 and 5–8 respectively. Tat(+) vehicle- and ZCZ011-treated mice are represented by lanes 9–12 and 13–16 respectively. M: molecular weights of marker protein (kDa). (TIF) [file pone.0305868.s003.tif]

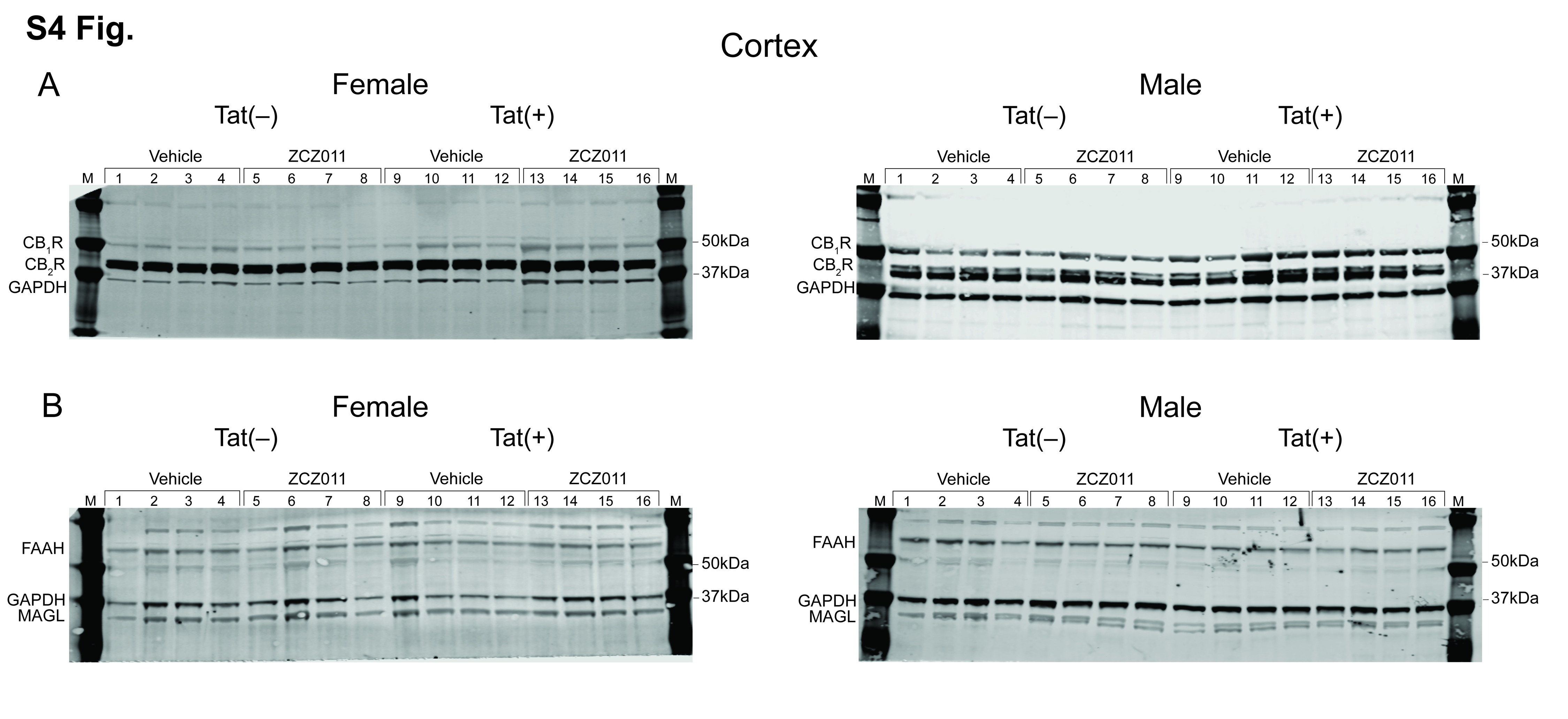

Supplement: S4 Fig — Images show original (A) CB1R, CB2R, and GAPDH and (B) FAAH, MAGL, and GAPDH for females and males. Tat(–) vehicle- and ZCZ011-treated mice are represented by lanes 1–4 and 5–8 respectively. Tat(+) vehicle- and ZCZ011-treated mice are represented by lanes 9–12 and 13–16 respectively. M: molecular weights of marker protein (kDa). (TIF) [file pone.0305868.s004.tif]

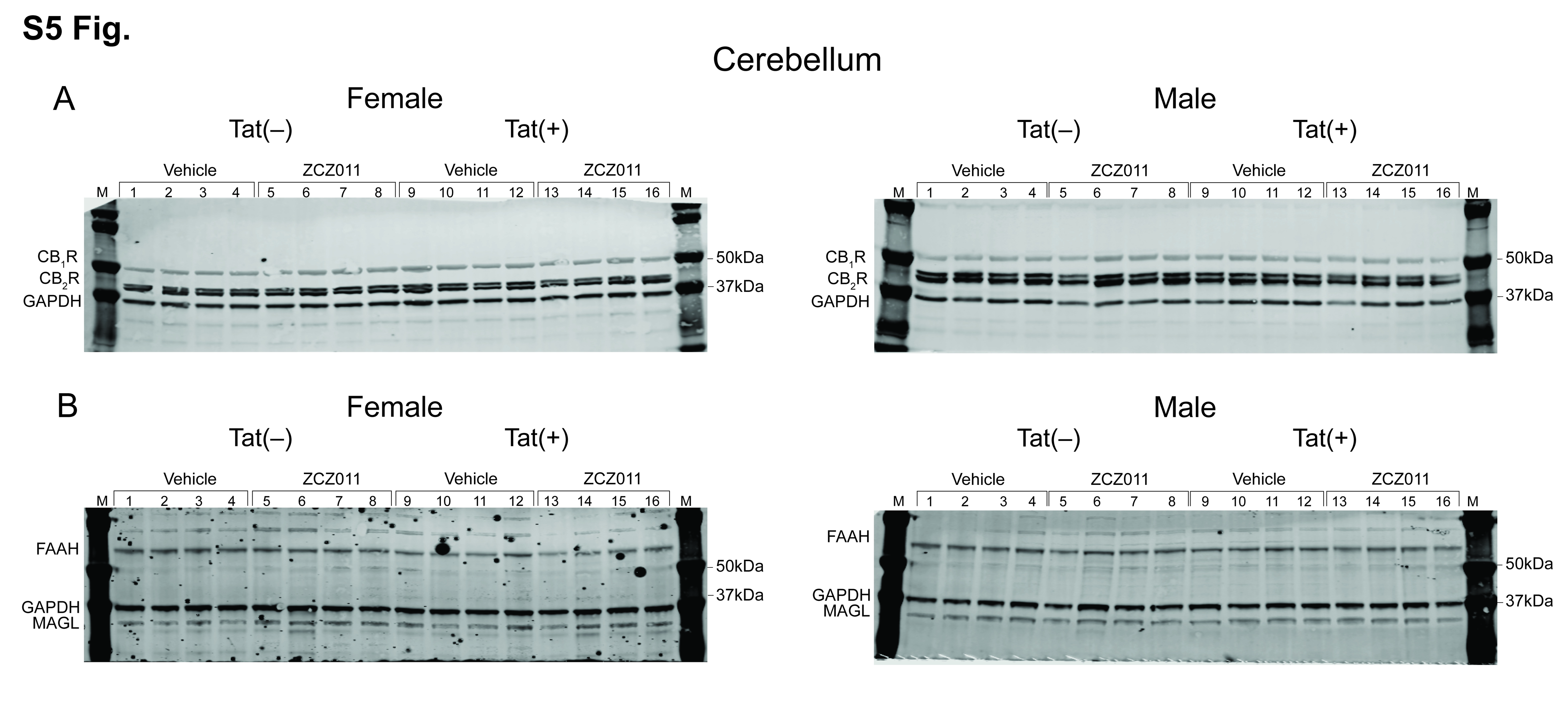

Supplement: S5 Fig — Images show original (A) CB1R, CB2R, and GAPDH and (B) FAAH, MAGL, and GAPDH for females and males. Tat(–) vehicle- and ZCZ011-treated mice are represented by lanes 1–4 and 5–8 respectively. Tat(+) vehicle- and ZCZ011-treated mice are represented by lanes 9–12 and 13–16 respectively. M: molecular weights of marker protein (kDa). Note: The specks seen in (B) was only seen in the green channel and therefore did not interfere with the quantification of FAAH or GAPDH. (TIF) [file pone.0305868.s005.tif]

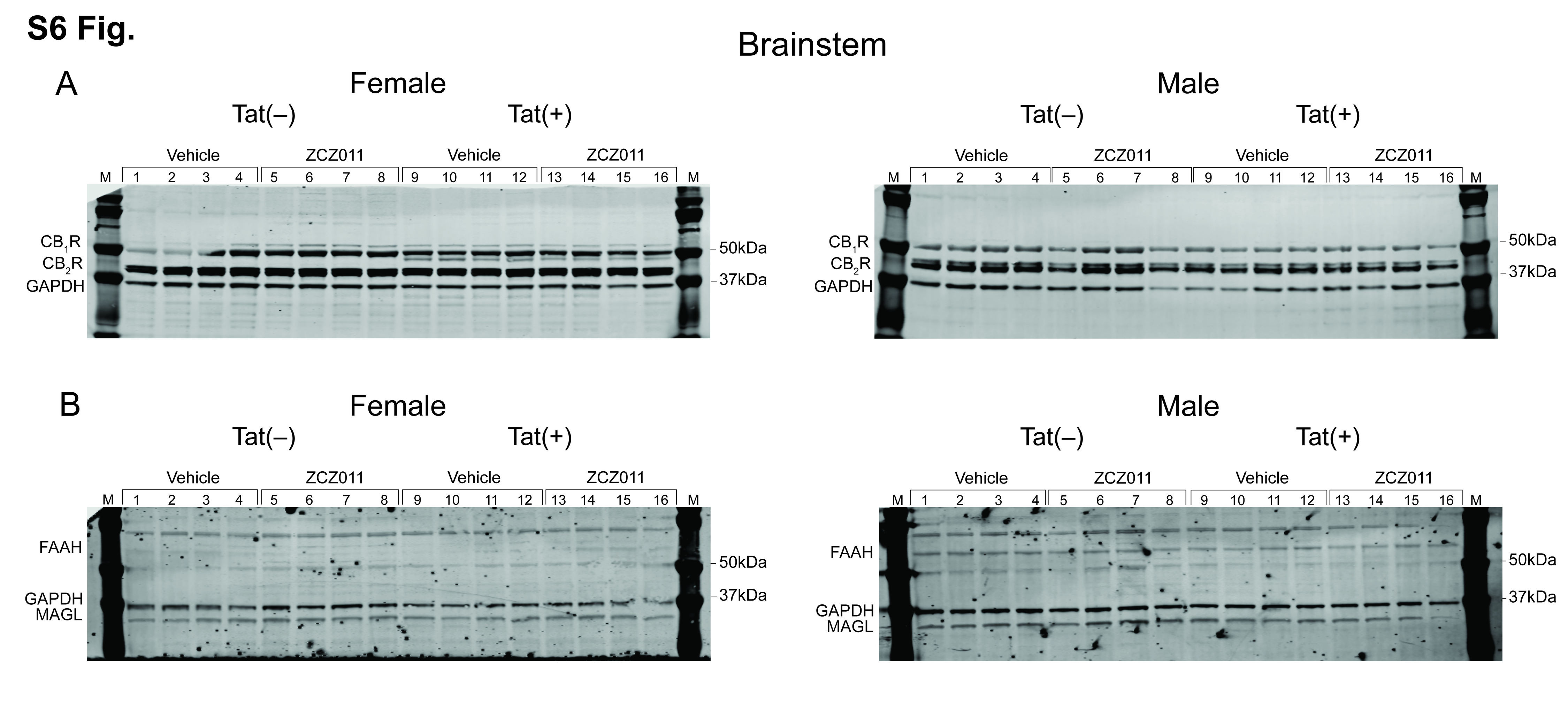

Supplement: S6 Fig — Images show original (A) CB1R, CB2R, and GAPDH and (B) FAAH, MAGL, and GAPDH for females and males. Tat(–) vehicle- and ZCZ011-treated mice are represented by lanes 1–4 and 5–8 respectively. Tat(+) vehicle- and ZCZ011-treated mice are represented by lanes 9–12 and respectively. M: molecular weights of marker protein (kDa). Note: The specks seen in (B) was only seen in the green channel and therefore did not interfere with the quantification of FAAH or GAPDH. (TIF) [file pone.0305868.s006.tif]

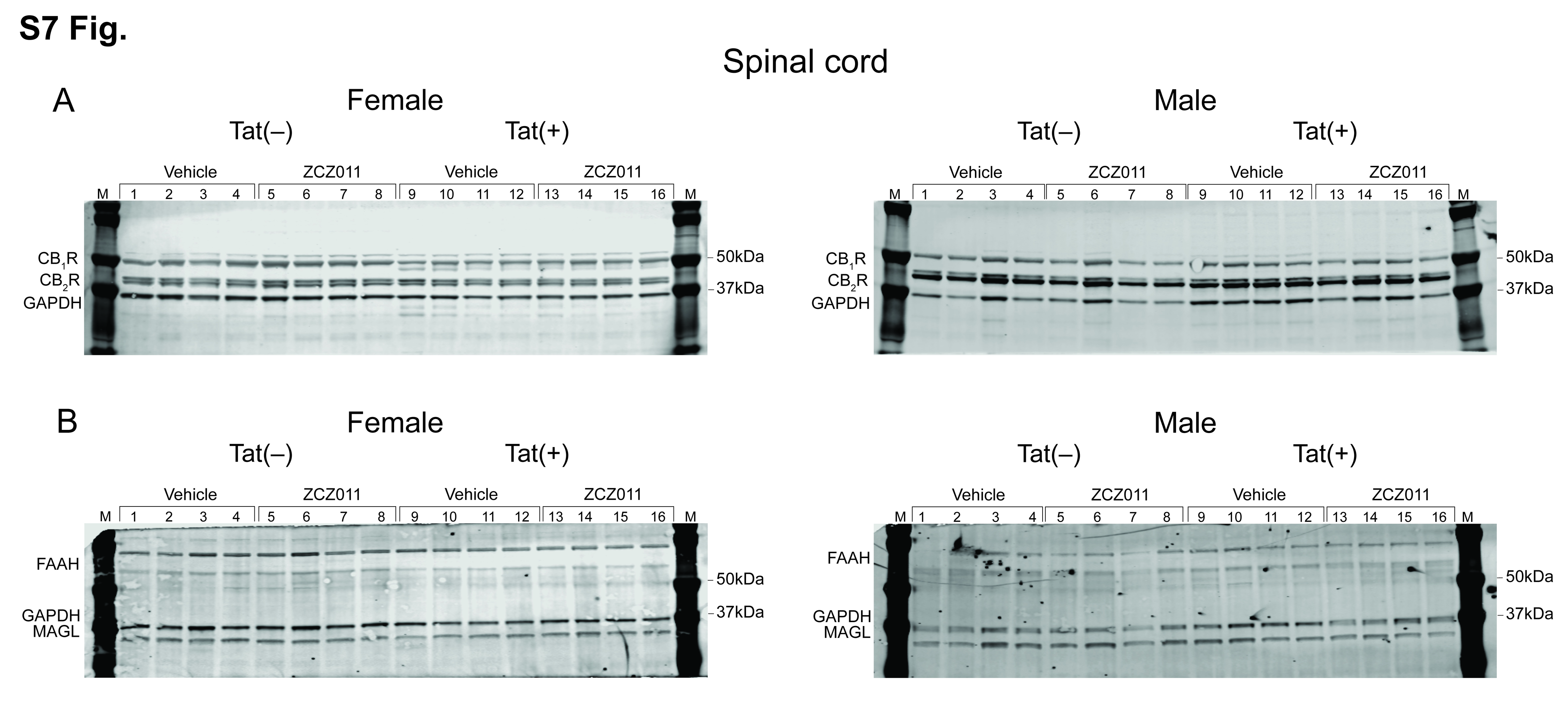

Supplement: S7 Fig — Images show original (A) CB1R, CB2R, and GAPDH and (B) FAAH, MAGL, and GAPDH for females and males. Tat(–) vehicle- and ZCZ011-treated mice are represented by lanes 1–4 and 5–8 respectively. Tat(+) vehicle- and ZCZ011-treated mice are represented by lanes 9–12 and 13–16 respectively. M: molecular weights of marker protein (kDa). (TIF) [file pone.0305868.s007.tif]
