# Supplementary material for: Cannabinoid receptor 1 positive allosteric modulator ZCZ011 shows differential effects on behavior and the endocannabinoid system in HIV-1 Tat transgenic female and male mice
Source: PLoS One. 2024 Jun 24;19(6):e0305868. doi: 10.1371/journal.pone.0305868 (PMC11195999; doi:10.1371/journal.pone.0305868)
Supplement: S1 Table — (PDF) [file pone.0305868.s008.pdf]

S1 Table

CNS Region	Lipids nm/mg	Sex	Genotype	Vehicle mean ± SEM	ZCZ011 mean ± SEM	Genotype Effect *p*	Sex Effect *p*	Drug Effect *p*	Genotype x Drug *p*	Genotype x Sex *p*	Sex x Drug p
PFC	AEA	Female	Tat (+)	0.06 ± 0.02	0.12 ± 0.04	0.84	0.89	0.29	0.12	0.27	0.30
			Tat (–)	0.10 ± 0.01	0.12 ± 0.04						
		Male	Tat (+)	0.09 ± 0.01	0.14 ± 0.04						
			Tat (–)	0.10 ± 0.03	0.06 ± 0.01						
	2-AG	Female	Tat (+)	3.56 ± 0.48	3.21 ± 1.00	0.34	0.84	0.41	0.10	0.95	0.18
			Tat (–)	1.44 ± 0.10	4.28 ± 1.57						
		Male	Tat (+)	3.63 ± 0.78	2.99 ± 0.65						
			Tat (–)	2.70 ± 0.55	2.73 ± 0.48						
	PEA	Female	Tat (+)	0.96 ± 0.24	1.67 ± 0.53	0.86	0.83	0.35	0.43	0.47	0.07
			Tat (–)	1.06 ± 0.12	1.88 ± 0.75						
		Male	Tat (+)	1.34 ± 0.23	1.58 ± 0.30						
			Tat (–)	1.58 ± 0.35	0.85 ± 0.08						
	OEA	Female	Tat (+)	0.68 ± 0.22	1.17 ± 0.33	0.65	0.99	0.32	0.37	0.25	0.11
			Tat (–)	0.80 ± 0.05	1.33 ± 0.51						
		Male	Tat (+)	1.03 ± 1.18	1.27 ± 0.25						
			Tat (–)	1.09 ± 0.26	0.60 ± 0.06						
	AA	Female	Tat (+)	401.18 ± 138.88	630.27 ± 196.22	0.23	0.81	0.27	0.22	0.29	0.25
			Tat (–)	422.21 ± 24.24	585.58 ± 138.63						
		Male	Tat (+)	498.14 ± 76.64	676.38 ± 172.73						
			Tat (–)	484.27 ± 128.88	299.19 ± 35.70						
Str	AEA	Female	Tat (+)	0.06 ± 0.02	0.05 ± 0.003	0.53	**0.004**	0.23	0.53	0.16	0.89
			Tat (–)	0.07 ± 0.01	0.06 ± 0.08						
		Male	Tat (+)	0.09 ± 0.10	0.09 ± 0.01						
			Tat (–)	0.08 ± 0.01	0.07 ± 0.005						
	2-AG	Female	Tat (+)	11.86 ± 5.32	14.16 ± 7.18	0.42	0.10	0.87	0.53	0.49	0.70
			Tat (–)	8.07 ± 5.17	8.99 ± 3.36						
		Male	Tat (+)	4.94 ± 0.68	6.95 ± 2.70						
			Tat (–)	7.32 ± 3.91	3.92 ± 0.41						
	PEA	Female	Tat (+)	1.60 ± 0.27	1.70 ± 0.27	0.30	**<0.001**	0.46	0.48	0.07	0.75
			Tat (–)	2.18 ± 0.40	1.55 ± 0.34						
		Male	Tat (+)	3.21 ± 0.25	3.11 ± 0.68						
			Tat (–)	2.46 ± 0.27	2.36 ± 0.13						
	OEA	Female	Tat (+)	1.28 ± 0.23	1.48 ± 0.25	0.32	**0.005**	0.62	0.46	0.08	0.80
			Tat (–)	1.78 ± 0.24	1.30 ± 0.33						
		Male	Tat (+)	2.39 ± 0.29	2.30 ± 0.46						
			Tat (–)	1.79 ± 0.12	1.79 ± 0.15						
	AA	Female	Tat (+)	501.07 ± 133.43	410.10 ± 22.41	0.55	**0.04**	0.12	0.86	0.43	0.69

			Tat (–)	533.64 ± 86.14	399.03 ± 56.91						
		Male	Tat (+)	655.84 ± 46.32	583.86 ± 115.32						
			Tat (–)	572.43 ± 66.42	508.45 ± 49.71						
Crb	AEA	Female	Tat (+)	0.04 ± 0.01	0.04 ± 0.003	0.35	0.32	**0.02**	**0.01**	0.06	0.89
			Tat (–)	0.04 ± 0.002	0.07 ± 0.02						
		Male	Tat (+)	0.04 ± 0.004	0.05 ± 0.004						
			Tat (–)	0.03 ± 0.004	0.05 ± 0.004						
	2-AG	Female	Tat (+)	11.29 ± 1.95	6.96 ± 0.85	0.29	0.24	0.29	**0.03**	**0.01**	0.43
			Tat (–)	9.67 ± 1.09	12.18 ± 1.49						
		Male	Tat (+)	17.73 ± 3.97	12.01 ± 1.80						
			Tat (–)	9.27 ± 3.08	9.34 ± 0.92						
	PEA	Female	Tat (+)	0.95 ± 0.21	0.66 ± 0.85	0.84	0.14	0.21	**0.03**	0.35	0.88
			Tat (–)	0.66 ± 0.13	1.24 ± 0.30						
		Male	Tat (+)	0.71 ± 0.26	0.75 ± 0.10						
			Tat (–)	0.47 ± 0.15	0.79 ± 0.11						
	OEA	Female	Tat (+)	1.29 ± 0.16	1.08 ± 0.09	0.57	0.33	**0.03**	**0.009**	0.11	0.94
			Tat (–)	1.08 ± 0.11	1.83 ± 0.36						
		Male	Tat (+)	1.23 ± 0.19	1.29 ± 0.08						
			Tat (–)	0.87 ± 0.14	1.39 ± 0.10						
	AA	Female	Tat (+)	706.06 ± 77.57	585.90 ± 65.28	0.09	**0.01**	0.29	**0.01**	**0.02**	0.72
			Tat (–)	731.06 ± 63.77	998.91 ± 107.82						
		Male	Tat (+)	654.94 ± 82.28	604.69 ± 44.53						
			Tat (–)	530.02 ± 68.15	655.24 ± 61.46						
SC	AEA	Female	Tat (+)	0.02 ± 0.004	0.03 ± 0.006	0.40	0.80	0.74	**0.05**	0.37	0.26
			Tat (–)	0.03 ± 0.003	0.02 ± 0.004						
		Male	Tat (+)	0.02 ± 0.002	0.03 ± 0.003						
			Tat (–)	0.04 ± 0.02	0.01 ± 0.003						
	2-AG	Female	Tat (+)	25.81 ± 5.66	26.34 ± 5.34	0.75	0.26	0.74	0.40	0.35	0.47
			Tat (–)	19.73 ± 1.83	22.35 ± 2.28						
		Male	Tat (+)	25.09 ± 6.73	28.64 ± 2.36						
			Tat (–)	35.36 ± 10.18	23.36 ± 5.46						
	PEA	Female	Tat (+)	2.50 ± 0.34	3.05 ± 0.72	0.62	0.78	0.65	0.09	0.10	0.25
			Tat (–)	2.22 ± 0.33	2.28 ± 0.44						
		Male	Tat (+)	1.65 ± 0.24	2.20 ± 0.23						
			Tat (–)	3.84 ± 1.37	1.90 ± 0.17						
	OEA	Female	Tat (+)	1.58 ± 0.18	2.21 ± 9.46	0.54	0.73	0.88	**0.05**	0.12	0.21
			Tat (–)	1.64 ± 0.22	1.62 ± 0.28						
		Male	Tat (+)	1.18 ± 0.17	1.56 ± 0.15						
			Tat (–)	2.54 ± 9.85	1.39 ± 0.89						
	AA	Female	Tat (+)	450.31 ± 51.83	596.88 ± 111.80	0.33	**0.01**	0.67	0.20	0.07	0.22
			Tat (–)	509.50 ± 57.85	447.97 ± 69.76						

		Male	Tat (+)	324.37 ± 37.38	269.74 ± 46.42						
			Tat (–)	504.92 ± 117.44	384.74 ± 50.25						

Levels of N-arachidonoylethanolamine (AEA), 2-arachidonoylglycerol (2-AG), palmitoylethanolamide (PEA), oleoylethanolamide (OEA), arachidonic acid (AA) in the prefrontal cortex, striatum, cerebellum, and spinal cord of Tat(–) and Tat(+) female and male mice exposed to chronic 10 mg/kg ZCZ011 or vehicle expressed as mean ± SEM. A three-way ANOVE for each lipid molecule was conducted with drug, genotype, and sex as between-subjects factors. Red bolded values denote significant differences at $p < 0.05$; $N = 32(16F)$.
