# Supplementary material for: Cannabinoid receptor 1 positive allosteric modulator ZCZ011 shows differential effects on behavior and the endocannabinoid system in HIV-1 Tat transgenic female and male mice
Source: PLoS One. 2024 Jun 24;19(6):e0305868. doi: 10.1371/journal.pone.0305868 (PMC11195999; doi:10.1371/journal.pone.0305868)
Supplement: S2 Table — (PDF) [file pone.0305868.s009.pdf]

S2 Table.

CNS Region	Receptors and enzymes	Sex	Genotype	Vehicle mean ± SEM	ZCZ011 mean ± SEM	Genotype Effect *p*	Sex Effect *p*	Drug Effect *p*	Genotype x Drug *p*	Genotype x Sex *p*	Sex x Drug *p*
PFC	CB_1R	Female	Tat (+)	0.40 ± 0.13	0.49 ± 0.08	0.17	0.85	0.25	0.16	0.07	0.17
			Tat (–)	0.54 ± 0.01	0.42 ± 0.04						
		Male	Tat (+)	0.44 ± 0.13	0.76 ± 0.20						
			Tat (–)	0.31 ± 0.04	0.39 ± 0.04						
	CB_2R	Female	Tat (+)	1.11 ± 0.12	0.65 ± 0.12	**0.04**	**0.02**	0.31	0.40	0.14	0.10
			Tat (–)	0.94 ± 0.16	0.86 ± 0.13						
		Male	Tat (+)	1.35 ± 0.39	2.22 ± 0.55						
			Tat (–)	1.00 ± 0.17	1.16 ± 0.14						
	FAAH	Female	Tat (+)	0.74 ± 0.10	0.54 ± 0.12	0.27	**0.03**	0.06	0.26	**0.01**	0.09
			Tat (–)	2.46 ± 0.76	0.78 ± 0.04						
		Male	Tat (+)	2.01 ± 0.42	1.81 ± 0.31						
			Tat (–)	1.44 ± 0.26	1.56 ± 0.31						
	MAGL	Female	Tat (+)	0.32 ± 0.01	0.33 ± 0.06	0.06	**<0.001**	**0.05**	**0.04**	**0.05**	**0.04**
			Tat (–)	1.15 ± 0.40	0.30 ± 0.02						
		Male	Tat (+)	0.03 ± 0.01	0.03 ± 0.004						
			Tat (–)	0.18 ± 0.006	0.02 ± 0.006						
Str	CB_1R	Female	Tat (+)	0.34 ± 0.04	0.50 ± 0.07	0.12	**<0.001**	0.96	**0.003**	0.45	0.61
			Tat (–)	0.45 ± 0.02	0.28 ± 0.02						
		Male	Tat (+)	0.11 ± 0.01	0.11 ± 0.01						
			Tat (–)	0.07 ± 0.11	0.11 ± 0.02						
	CB_2R	Female	Tat (+)	0.97 ± 0.02	1.27 ± 0.12	0.46	**<0.001**	0.61	0.08	0.38	0.47
			Tat (–)	1.28 ± 0.06	0.96 ± 0.09						
		Male	Tat (+)	0.74 ± 0.04	0.74 ± 0.08						
			Tat (–)	0.55 ± 0.07	0.72 ± 0.14						
	FAAH	Female	Tat (+)	0.20 ± 0.02	0.18 ± 0.02	0.93	**<0.001**	0.91	**<0.001**	**0.03**	0.11
			Tat (–)	0.25 ± 0.01	0.22 ± 0.04						
		Male	Tat (+)	0.24 ± 0.01	0.48 ± 0.04						
			Tat (–)	0.40 ± 0.004	0.24 ± 0.03						
	MAGL	Female	Tat (+)	0.09 ± 0.01	0.09 ± 0.01	0.78	**<0.001**	0.46	**0.03**	0.15	0.64
			Tat (–)	0.06 ± 0.006	0.07 ± 0.01						
		Male	Tat (+)	0.13 ± 0.01	0.21 ± 0.02						
			Tat (–)	0.20 ± 0.03	0.16 ± 0.03						
Hip	CB_1R	Female	Tat (+)	0.19 ± 0.01	0.13 ± 0.003	0.14	**<0.001**	**<0.001**	**0.04**	**0.002**	**0.04**
			Tat (–)	0.21 ± 0.03	0.18 ± 0.03						
		Male	Tat (+)	0.47 ± 0.04	0.29 ± 0.04						

			Tat (–)	0.29 ± 0.01	0.27 ± 0.03						
	CB_2R	Female	Tat (+)	0.99 ± 0.05	0.82 ± 0.15	0.91	**<0.001**	**0.008**	0.53	**0.01**	0.78
			Tat (–)	1.23 ± 0.13	0.96 ± 0.09						
		Male	Tat (+)	0.87 ± 0.07	0.54 ± 0.04						
			Tat (–)	0.52 ± 0.04	0.48 ± 0.08						
	FAAH	Female	Tat (+)	0.65 ± 0.16	0.64 ± 0.06	0.53	0.39	0.13	0.60	0.30	0.79
			Tat (–)	1.14 ± 0.21	0.22 ± 0.04						
		Male	Tat (+)	1.14 ± 0.24	0.80 ± 0.21						
			Tat (–)	1.03 ± 0.47	0.76 ± 0.19						
	MAGL	Female	Tat (+)	0.59 ± 0.13	0.71 ± 0.04	0.63	0.95	0.68	0.81	0.74	0.40
			Tat (–)	0.66 ± 0.12	0.07 ± 0.01						
		Male	Tat (+)	0.80 ± 0.13	0.55 ± 0.10						
			Tat (–)	0.58 ± 0.24	0.61 ± 0.10						
Ctx	CB_1R	Female	Tat (+)	0.07 ± 0.01	0.07 ± 0.006	**0.01**	**<0.001**	0.21	0.42	**0.002**	**0.006**
			Tat (–)	0.09 ± 0.02	0.06 ± 0.01						
		Male	Tat (+)	0.23 ± 0.01	0.26 ± 0.02						
			Tat (–)	0.17 ± 0.01	0.21 ± 0.01						
	CB_2R	Female	Tat (+)	1.71 ± 0.34	1.15 ± 0.11	**0.01**	**<0.001**	0.23	0.34	**0.02**	0.29
			Tat (–)	2.2 ± 0.36	2.12 ± 0.26						
		Male	Tat (+)	0.63 ± 0.03	0.56 ± 0.06						
			Tat (–)	0.59 ± 0.06	0.64 ± 0.05						
	FAAH	Female	Tat (+)	2.58 ± 0.70	0.89 ± 0.14	0.97	**0.008**	0.64	0.18	0.93	0.77
			Tat (–)	1.20 ± 0.33	2.21 ± 1.60						
		Male	Tat (+)	0.37 ± 0.04	0.43 ± 0.02						
			Tat (–)	0.56 ± 0.09	0.35 ± 0.04						
	MAGL	Female	Tat (+)	2.12 ± 0.57	0.81 ± 0.12	0.73	**<0.001**	0.19	0.16	0.69	0.19
			Tat (–)	1.24 ± 0.28	1.36 ± 0.62						
		Male	Tat (+)	0.15 ± 0.01	0.23 ± 0.04						
			Tat (–)	0.24 ± 0.01	0.16 ± 0.02						
Crb	CB_1R	Female	Tat (+)	0.27 ± 0.01	0.27 ± 0.01	0.55	**<0.001**	0.62	0.57	**0.02**	0.43
			Tat (–)	0.36 ± 0.02	0.29 ± 0.03						
		Male	Tat (+)	0.14 ± 0.03	0.18 ± 0.07						
			Tat (–)	0.08 ± 0.01	0.16 ± 0.01						
	CB_2R	Female	Tat (+)	0.90 ± 0.03	0.90 ± 0.06	0.34	0.98	0.35	0.61	0.21	0.18
			Tat (–)	1.18 ± 0.09	1.09 ± 0.04						
		Male	Tat (+)	0.99 ± 0.20	1.08 ± 0.34						
			Tat (–)	0.79 ± 0.10	1.20 ± 0.04						
	FAAH	Female	Tat (+)	0.29 ± 0.02	0.24 ± 0.02	**0.01**	**<0.001**	0.26	**0.03**	0.56	0.65
			Tat (–)	0.49 ± 0.02	0.33 ± 0.04						
		Male	Tat (+)	0.54 ± 0.07	0.74 ± 0.09						
			Tat (–)	1.01 ± 0.17	0.72 ± 0.17						

	MAGL	Female	Tat (+)	0.06 ± 0.002	0.06 ± 0.002	**0.01**	**<0.001**	0.71	0.08	0.08	0.70
			Tat (–)	0.07 ± 0.005	0.07 ± 0.003						
		Male	Tat (+)	0.14 ± 0.02	0.20 ± 0.03						
			Tat (–)	0.29 ± 0.04	0.21 ± 0.06						
BS	CB_1R	Female	Tat (+)	0.39 ± 0.03	0.39 ± 0.09	0.57	0.43	0.47	**0.05**	0.40	0.91
			Tat (–)	0.13 ± 0.03	0.29 ± 0.02						
		Male	Tat (+)	0.54 ± 0.21	0.22 ± 0.06						
			Tat (–)	0.14 ± 0.03	0.68 ± 0.43						
	CB_2R	Female	Tat (+)	1.11 ± 0.05	1.17 ± 0.33	0.669	0.32	0.70	0.08	0.48	0.84
			Tat (–)	0.65 ± 0.02	0.74 ± 0.02						
		Male	Tat (+)	1.82 ± 0.64	0.69 ± 0.19						
			Tat (–)	0.56 ± 0.11	2.15 ± 1.35						
	FAAH	Female	Tat (+)	0.07 ± 0.02	0.33 ± 0.08	0.29	**0.01**	0.54	0.06	**0.05**	0.25
			Tat (–)	0.28 ± 0.03	0.02 ± 0.01						
		Male	Tat (+)	0.25 ± 0.03	0.19 ± 0.06						
			Tat (–)	0.24 ± 0.04	0.23 ± 0.08						
	MAGL	Female	Tat (+)	0.30 ± 0.02	0.36 ± 0.03	**0.001**	**<0.001**	**0.01**	0.11	0.54	0.52
			Tat (–)	0.07 ± 0.005	0.25 ± 0.04						
		Male	Tat (+)	0.22 ± 0.03	0.29 ± 0.02						
			Tat (–)	0.14 ± 0.02	0.23 ± 0.04						
SC	CB_1R	Female	Tat (+)	0.39 ± 0.09	0.47 ± 0.04	0.94	**<0.001**	0.55	0.40	**<0.001**	0.25
			Tat (–)	0.24 ± 0.02	0.31 ± 0.01						
		Male	Tat (+)	0.09 ± 0.02	0.13 ± 0.02						
			Tat (–)	0.31 ± 0.10	0.23 ± 0.04						
	CB_2R	Female	Tat (+)	0.68 ± 0.09	0.63 ± 0.04	0.27	**0.006**	0.94	0.58	**0.002**	0.94
			Tat (–)	0.34 ± 0.04	0.42 ± 0.02						
		Male	Tat (+)	0.50 ± 0.06	0.69 ± 0.14						
			Tat (–)	1.21 ± 0.35	1.02 ± 0.20						
	FAAH	Female	Tat (+)	0.10 ± 0.07	0.18 ± 0.04	**0.006**	**<0.001**	0.66	0.24	0.11	0.64
			Tat (–)	0.28 ± 0.06	0.19 ± 0.02						
		Male	Tat (+)	0.34 ± 0.03	0.47 ± 0.16						
			Tat (–)	0.72 ± 0.12	0.71 ± 0.15						
	MAGL	Female	Tat (+)	0.31 ± 0.07	0.36 ± 0.03	**0.03**	**<0.001**	0.21	0.28	**<0.001**	0.21
			Tat (–)	0.19 ± 0.04	0.14 ± 0.03						
		Male	Tat (+)	0.35 ± 0.12	0.34 ± 0.10						
			Tat (–)	0.68 ± 0.10	1.20 ± 0.32						

Levels of cannabinoid type 1 and 2 receptors (CB_1R and CB_2R) and degradative enzymes fatty acid amide hydrolase (FAAH) and monoacylglycerol (MAGL in the prefrontal cortex, striatum, hippocampus, cortex, cerebellum, brainstem, and spinal

cord of Tat(–) and Tat(+) female and male mice exposed to chronic 10 mg/kg ZCZ011 or vehicle expressed as mean ± SEM. A three-way ANOVE for each protein was conducted with drug, genotype, and sex as between-subjects factors. Red bolded values denote significant differences at p <0.05; N = 32(16F).
